# Thin Objects Are Not Transparent

**DOI:** 10.1111/theo.12373

**Published:** 2021-11-25

**Authors:** Matteo Plebani, Luca San Mauro, Giorgio Venturi

**Affiliations:** ^1^ Dipartimento di Filosofia e Scienze dell'Educazione Università degli Studi di Torino; ^2^ Dipartmento di Matematica Sapienza Università di Roma; ^3^ Departamento de Filosofia (IFCH) Universidade Estadual de Campinas (UNICAMP)

**Keywords:** thin objects, abstraction, Øystein Linnebo, Rice's theorem

## Abstract

In this short paper, we analyse whether assuming that mathematical objects are “thin” in Linnebo's sense simplifies the epistemology of mathematics. Towards this end, we introduce the notion of transparency and show that not all thin objects are transparent. We end by arguing that, far from being a weakness of thin objects, the lack of transparency of some thin objects is a fruitful characteristic mark of abstract mathematics.

## 1 Introduction

An object is thin when “little or nothing is required” (Linnebo, 2018, p. xi) for its existence. Linnebo's book (Linnebo, 2018) is a remarkably lucid and philosophically sophisticated attempt to clarify the notion of thin object and defend the view that mathematical objects are thin. As Linnebo acknowledges, the idea that mathematical objects are thin “holds great philosophical promise. If the existence of certain objects does not make a substantial demand on the world, then knowledge of such objects will be comparatively easy to attain. … [It] might well be the only way to reconcile the need for an ontology of mathematical objects with the need for a plausible epistemology” (Linnebo, 2018, p. xi).

In this paper, we want to critically examine whether such a promise has been maintained. The answer will be that it has been maintained only partially: even assuming that mathematical objects are thin, our knowledge of mathematical objects would still be problematic in many respects. For instance, the doctrine of thin objects might make it easy to prove that mathematical objects *a* and *b* exist but hard to know whether they are the same object or not. However, we argue, this is not a problem but rather a result to welcome.

## 2 An Epistemology for Mathematical Objects

One of the main reasons for arguing that mathematical objects are thin is the direct comparison with objects that, on the contrary, are thick. By thick objects, we mean those entities that need substantial assumptions for their existence. In principle, assumptions are not inconvenient, but they can become so when we deal with objects that are nonspatial, atemporal, and causally inert. As a matter of fact, the abstract character of mathematical objects leave the assumptions on their existence outside the scope of our experience. This leads to the creation of an insurmountable gap between the ontology and the epistemology of mathematical objects. Indeed, if mathematical sentences talk about mathematical objects whose existence is independent of us, how can we get any knowledge *from* them – or even realize that what we have is knowledge *of* them?

The advantages of thin objects over thick objects lie in the possibility to avoid choosing between an homogeneous semantic theory – for both mathematical and non‐mathematical language – and a reasonable epistemology for mathematics (as required by Benacerraf's problem [Benacerraf, 1973]). As a matter of fact, if mathematical objects are thin, then any epistemology that we find reasonable and that helps us navigate the mathematical world would comply more easily with the very light (if ever) requirements imposed on the existence of mathematical objects. In this way, the dilemma between ontology and epistemology is dispelled, and the scales are tipped in favour of epistemology simply by lighting the side of mathematical ontology.

But what kind of epistemology does this tipping the scales produces? Is the notion of thin object able to produce a reasonable epistemology for mathematics? In order to address this problem^1^ we may, ask ourselves what we expect from an epistemology of mathematics, understood as a science of mathematical objects. As an example of the complexity of this issue, consider a famous passage from Frege in which he indicates an important requirement for reference to *objects*:

If we are to use the symbol *a* to signify an object, we must have a criterion for deciding in all cases whether *b* is the same as *a*, *even if it is not always in our power to apply this criterion*. (Frege, 1884, §62) (our emphasis).

In this paper, we will show how important it is to remember the last part of the quotation above: we will present cases where the existence of abstract objects *a* and *b* can be easily established but where we have no mechanical procedure to establish whether *a* equals *b* or not. As will become clear, we do not think that the undecidability of some identities between thin objects is a problem for Linnebo's account, but it is an important point to keep in mind.

The view that mathematics is a science of objects is constitutive of a modern picture of mathematics.^2^ And many of the most challenging questions of the contemporary philosophy of mathematics are, in fact, related to this objects‐driven picture of mathematics. Thin objects are no exception. Their merit, indeed, can be found exactly in trying to explain away many puzzles related to the view that mathematics is a science of *sui generis* objects. The requirements we impose on a reasonable epistemology of mathematical objects, thus, bring us to the notion of *transparency*.

## 3 Thin Versus Transparent

Linnebo defines an object to be *thin* when “little or nothing is required” (Linnebo, 2018, p. xi) for its existence.

In particular, for many thin objects all that is required for their existence is the existence of certain unproblematic objects: the existence of lines is sufficient for the existence of directions, the existence of letter tokens is sufficient for the existence of letter types, and so on. Let us grant, for the sake of the argument, that when an object is thin it is easy to know that it exists.

Define an object *o* to be *transparent* if it is easy to recognize the “unproblematic” objects whose existence is sufficient for the existence of *o*.^3^ What interests us here is the relation between the notions of thinness and transparency. For now, let us state an important claim that we will later prove (see §3.1 below) in a formalized version.

**Claim:** *Not all thin objects are transparent*.

To state more precisely and prove our result, we need to make some assumptions. We assume that thin objects are obtained by a process of abstraction over a domain of non‐problematic objects *D*, the *base domain*, according to an abstraction principle of this form (where *α* and *β* are elements of *D* and ∼ is an equivalence relation over *D*, called the *unity relation*):

$$f\;\left(\alpha \right)=f\;\left(\beta \right)\;↔\;\alpha \;∼\;\beta .$$

We identify the set of unproblematic objects that are sufficient for the existence of the thin object *f*(*α*) with {*x* ∈ *D*: *x* ∼ *α*}. We call the elements of {*x* ∈ *D*: *x* ∼ *α*} the *names* or the *specifications* of *f*(*α*). By saying that the elements of {*x* ∈ *D*: *x* ∼ *α*} can be easily recognized, we mean that {*x* ∈ *D*: *x* ∼ *α*} is computable; if such a set is computably enumerable, we say that the corresponding object is *semi‐transparent*.

Observe that the notion of transparency is intertwined with the decidability of the *identity problem* for thin objects (as presented by some specifications). That is, opaque thin objects are exactly those for which it is undecidable to say whether *f*(*α*) = *f*(*β*) – even though it is unproblematic to decide if *α* equals *β*.

Here is an example of a class of transparent thin objects. Take ℤ as *D* and congruence modulo *n* as ∼. The thin objects generated by the abstraction principle are transparent because there is an effective procedure to establish, given an integer modulo *n*, whether an integer is one of its names or not.

Here is an example of non‐transparent, or *opaque*, thin objects. Take ℕ as the base domain and let ∼ be the equivalence relation that holds between *m* and *n* just in case both numbers are the codes (for a suitable coding *à la* Gödel) of theorems of first‐order logic or none of them are.

This second example shows that the claim that there are thin objects that are not transparent is, in a sense, obvious and unexciting. However, in what follows we will prove a more significant version of the above claim: for some *interesting* choices of *D* and ∼, none of the thin objects introduced by the relevant abstraction principle are transparent.

As an illustration, consider the case where *D* is the set of the Turing machines programs (conceived here as concrete tokens, say, sequences of marks on a surface) and the equivalence relation is the one that holds between two programs just in case for any (code of a) numerical input they return the same output. The thin objects introduced by the abstraction principle operating on that base domain and that equivalence relation are the (partial) computable functions on ℕ: it can be proved that *none* of these thin objects are transparent nor semi‐transparent (see theorem 3.1).

A clarification is in order here: if the base domain is finite, all relations are decidable and every abstract object is transparent. Hence, the existence of opaque thin objects requires the base domain to be infinite.

We believe that the assumption of an infinite base domain is compatible with ontological minimalism (the thesis that mathematical objects are thin). One of the most famous examples of a base domain is the set of lines: why should we assume that there are finitely many lines? The abstraction principle $D\;\left(a\right)=D\;\left(b\right)\;↔\;a∥b$ seems to be compatible both with the assumption that there are finitely many lines and the assumption that there are infinitely many lines.

Moreover, our results can be reformulated assuming that the base domain is *potentially* infinite. This is an assumption that ontological minimalists should be happy to concede. One of Linnebo's favourite examples of a base domain is a set of tokens (Linnebo, 2018, ch. 8 and 10). (Numeral) tokens, according to Linnebo, are potentially infinite: given any list of numerals, there might be a numeral longer than any of them. Any program for a Turing machine can be identified with a possible numeral token. If the base domain and the unity relation are defined as above, we can reformulate our result in this way: for any abstract object, the problem whether any two possible tokens are specifications of that abstract object is undecidable; moreover, for any abstract object, there is no mechanical procedure to list all of its *possible* specifications. Hence, our results still obtain even on the assumption that the base is domain is only potentially infinite.

### 3.1 Formal results

Here, we collect two explanatory results which serve to illustrate and support our thesis on the interaction between thinness and transparency. The first theorem, which we already informally sketched, is a reformulation of Rice's theorem – a cornerstone of classical computability theory (see, e.g., Soare, 2016, Thm. 1.6.14). It illustrates that thin objects can be non‐transparent. The second theorem, which builds on Pour‐El and Kripke (1967) and Bernardi and Sorbi (1983), shows that thin objects emerging from a natural unity relation –namely, provable equivalence of arithmetic formulas – are all semi‐transparent but not transparent.

Theorem 3.1 Let *X* be a collection of thin functions g:ℕ→ℕ which are abstracted from a base domain *D*_*X*_ equipped a unity relation ∼X. If

1. $D_{X}⊇ℳ$, where ℳ is the class of all Turing machines;
2. and $∼X{↾}_{ℳ}$ coincides with the equivalence relation which equate Turing machines if and only if they compute the same functions,

then no object in *X* is transparent.

Proof. To prove that any function *g* ∈ *X* is opaque, we have to show that the set of *g*‐specifications is not computable. Suppose otherwise. We will reach a contradiction by showing that then the Halting problem would be also computable. As usual, we denote by {*ϕ*_*e*_}_*e*∈*ω*_ a uniform enumeration of all (partial) computable functions.

Denote by *λ* the completely undefined function. We distinguish two cases. First, suppose that *g* ≠ *λ*. Let *A*_*e*_ be the Turing machine which, on any input *n*, performs the following instructions,

- Execute *ϕ*_*e*_.
- If the above computation terminates, output *g*(*n*).

Note that, if *ϕ*_*e*_(*e*) halts, then *A*_*e*_ computes *g*, in which case, by items (1) and (2) above, *A*_*e*_ must be a *g*‐specification. Otherwise, *ϕ*_*e*_(*e*) diverges, *A*_*e*_ computes *λ*. Hence, we have that

$$\phi_{e}\left(e\right)\;\text{halts if and only if}\;A_{e}\;\text{is}\;a\;g‐\text{specification}.$$

So, the set of *g*‐specifications cannot be computable because otherwise one could decide the Halting problem.

On the other hand, suppose that *g* = *λ*. If so, fix a computable function *h* ≠ *λ*. Recall that a set is computable if and only if its complement is computable. So, it suffices to prove that the complement of the set of *g*‐specifications is noncomputable. By reasoning as above relatively to *h* (i.e., by letting Turing machines computing *h* only if *ϕ*_*e*_(*e*) halts), one immediately constructs *B*_*e*_'s Turing machines so that

$$\phi_{e}\left(e\right)\;\text{halts if and only if}\;B_{e}\;\text{is}\;\text{not}\;a\;g‐\text{specification}.$$

Hence, the complement set of the *g*‐specifications cannot be computable, as desired. □

It is not difficult to see that none of the thin functions considered in the last theorem are even semi‐transparent. The next theorem offers a natural collection of thin objects which are semi‐transparent and opaque.

Theorem 3.2 Let *D* be the base domain of (first‐order) arithmetic formulas, and let ∼PA be the unity relation which equates *ϕ*, *ψ* ∈ *D* if and only

PA⊢ϕ↔ψ,

where *PA* denotes Peano Arithmetic. If *PA* is consistent, then none of the thin objects obtained by abstracting over *D* and ∼PA are transparent, but all of them are semi‐transparent.

Proof. Denote by **0** and **1** the objects obtained by abstracting from the anti‐theorems and the theorems of *PA*, respectively. It is a clear consequence of Gödel's incompleteness that neither **0** nor **1** are transparent because, for example, {*α* ∈ *D*: *α* $\sim_{\text{PA}}$ 0 = 0} is not computable. In fact, all other equivalence classes of ∼_*PA*_ are also noncomputable. This is because, given *α*, *β* ∈ *D*, we have that

$$x\in {\left[\alpha \right]}_{\sim_{\mathrm{PA}}}\;\text{if and only if}\;\left(\beta ↔\left(x↔\alpha \right)\right)\in {\left[\beta \right]}_{\sim_{\mathrm{PA}}}.$$

Therefore, all $\sim_{\text{PA}}$‐classes are pairwise computably isomorphic, and thus none are computable. It follows that no object corresponding to one of these classes is transparent. On the other hand, all of them are semi‐transparent because any ${\left[\alpha \right]}_{\sim_{\mathrm{PA}}}$ is defined as $\left\{\beta :\mathrm{PA}⊢\alpha ↔\beta \right\}$, which is clearly computably enumerable. □

Of course, the last theorem applies to any first‐order theory for which Gödel's theorems hold.

## 4 The Significance of Opacity

Why should an ontological minimalist care about the fact that some thin objects fail to be transparent? Are we suggesting that ontological minimalism is incompatible with the fact that the elements of some mathematically interesting class of thin objects are all opaque? We are not and, indeed, we are happy to grant that in principle an ontological minimalist might not be moved by what we argued so far.

However, our discussion of opaque thin objects might clarify how to correctly interpret certain claims made by ontological minimalists when advertising their doctrine. Recall how ontological minimalism is supposed to help to account for the accuracy of our beliefs about thin objects:

Directions are specified by means of lines, which are assumed to be unproblematic. And since all the properties and relations of directions are “inherited” from corresponding properties of lines, they don't pose any additional epistemological problem. (Linnebo, 2017, p. 128)

Our discussion of opaque thin objects invites caution in evaluating claims like this. The elements of the base domain might be “unproblematic” in the sense that their existence is not under discussion. A certain equivalence relation on the base domain (the unity relation) might be unproblematic in the sense that we recognize clear cases of pairs of objects that stand in such a relation and clear cases of pairs of objects that do not stand in such a relation. This allows us to expand the domain introducing a class of abstract objects via a principle of abstraction based on the unity relation. That the base domain is unproblematic in this sense does not guarantee that the unity relation be decidable.

Actually, if we want to obtain interesting mathematical objects like the (partial) computable functions on ℕ via abstraction on the base domain, there is reason to think that the unity relation cannot be decidable. This in turn entails that the abstract objects introduced are *opaque*: although for many of those objects it might be easy to know that they do have *some* specifications, there is no mechanical criterion to establish – given an abstract object and element of the base domain – whether the element of the base domain is one of the specifications of the abstract object.

One might reply that all of this is consistent with Linnebo's claim in the quote above that abstract objects introduced via an abstraction principle “don't pose any *additional* epistemological problem” (Linnebo, 2017, p. 128; our emphasis): if abstract objects are opaque, that is the case only because the unity relation is undecidable.

It is true that the reason why abstract objects are opaque is that the unity relation is undecidable. However, our discussion of opaque thin objects offers a new perspective on the thesis that the method of abstraction provides clear *identity criteria* for the abstract objects that it introduces. Stipulating that $f\left(\alpha \right)=f\left(\beta \right)↔\alpha ∼\beta$ reduces the problem of whether *f*(*α*) = *f*(*β*) to the problem of whether *α* ∼ *β*, but it also entails that as long as the unity relation is undecidable, the same goes for identity problem for abstract objects.

To repeat: that certain identities between thin objects are undecidable does not entail that thin objects do not exist or that we cannot attain knowledge of them. However, reflecting on the fact that some thin objects are opaque is important to appreciate the qualification made by Frege when discussing criteria of identity in §62 of (Frege, 1884); sometimes it might not be “within our powers to apply” the identity criterion for a certain class of thin objects.

In other words, stipulating that $f\left(\alpha \right)=f\left(\beta \right)↔\alpha ∼\beta$ might *increase* the complexity of the identity problem: when the identity problem over the base domain is decidable and the unity relation is not, the identity problem over abstract objects ends up being more complex than the identity problem over the base domain.^4^ Assuming that the base domain is composed of concrete objects, like linguistic tokens, that are not the result of an abstraction process, then the objects of the base domain are all transparent, both in a trivial sense (given that the objects of the base domain are not the result of an abstraction process, they do not have any specification; hence the class of their specification is empty and therefore computable) and in the more interesting sense that we have an algorithm to answer questions of the form “*α* = *β*?” Expanding the domain through abstraction, we encounter objects of a different kind: the objects of the expanded domain are no longer transparent.

The opacity of some thin objects also relates to the famous *Kreisel dictum*:

As Kreisel remarked in a review of Wittgenstein, “the problem is not the existence of mathematical objects but the objectivity of mathematical statements.” (Dummett, 1978, p. 38)

Here is how the presence of opaque thin objects is connected with the problem of the objectivity of our mathematical statements, the problem whether to assume that any mathematical statement has a truth value independently of our ability to determine which. If all thin objects were transparent, then we would always be in a position to answer questions of the form “*f*(*α*) = *f*(*β*)?”; however, if *f*(*α*) is an opaque thin object, we might not be able to answer to whether *f*(*α*) = *f*(*β*) or not. In the latter case, should we assume that the question has an answer despite our inability to find it or not? The fact that some thin objects are opaque forces us to face this kind of questions.

Linnebo explicitly states that ontological minimalism is neutral with respect to the question of objectivity (Linnebo, 2018, ch. 11.5).^5^ Indeed, ontological minimalism is compatible both with the thesis that mathematical statements have an objective truth value independently of our ability to determine it and with the thesis that undecidable mathematical statements lack a truth value. However, this shows precisely that ontological minimalism leaves some hard epistemological questions unanswered. Knowing that the thin objects *f*(*α*) and *f*(*β*) exist might be easy. Knowing the answer to whether *f*(*α*) = *f*(*β*) might in some cases be hard, and one might even wonder whether the question has a determinate answer.

In the end, our main point is one that Linnebo himself seems to acknowledge:

[S]ome of the hardest problems in the epistemology of mathematics derive, not from its ontology of abstract objects […] While Benacerraf's problem is certainly important, it is unfortunate that the great amount of attention it has received has come at the expense of other, equally important problems in the epistemology of mathematics. (Linnebo, 2018, p. 4)

The problem whether identity claims about opaque thin objects have an objective truth value is an example of the kind of problems that remain open even if we grant that ontological minimalism offers a solution to Benacerraf's problem.

## 5 An Optimistic Moral

In this short paper, we argued that the light ontology of thin objects comes at a cost: an opaque epistemology for mathematics. In this respect the meta‐ontological minimalist is in good company because she is facing what is a problem, as well, for the realist. The meta‐ontological minimalist scores better with respect to ontology, but she is consistent with the results of the realist for what concerns epistemology. Should we interpret this conclusion as a weakness of thin objects, or does this observation only suggest a common trait of both realism and meta‐ontological minimalism?

We think that the latter is the case and that we should not be afraid of opacity. Indeed, what might look as a deficiency of the underlying epistemology of mathematics can be seen, on the contrary, as a strength of the language of mathematics.

Although thin objects allow for a uniform semantics for mathematical and non‐mathematical language, we suggested that this achievement does not necessarily produce a transparent epistemological access to the objects of mathematics. However, the opacity of thin objects is exactly what allows abstraction principles to offer a higher perspective on their subject matter. As a matter of fact, the opacity that ensues from abstraction principles cannot be separated from the abstract perspective they offer on mathematical objects. The possibilities provided by abstraction principles consist in producing representatives of entire classes of mathematical objects and in capturing common treats of possibly infinitely many elements of a domain *D*. These are the characteristic marks of the simplification they introduce in mathematics. The relation between the simple elements of *D* and the complex *abstracta* that result from an abstraction principle is, in practice, reversed by the mathematical fruitfulness that abstraction offers in dealing with objects that escape our sensory grasp. In other terms, the connection between a non‐problematic domain of (mathematical) objects and a problematic range of *abstracta* is, from the perspective of mathematical practice, a tool for accessing a new realm of objects that is able to simplify (due to their abstract character) our general understanding of mathematics. This happens at the expense of our understanding of mathematical ontology; however, we believe that this is a necessary cost we have to pay to gain mathematical knowledge. The expressivity that abstraction principles offer to mathematical language, together with the most celebrated abstract character of mathematics, is exactly what allows mathematicians the right distance from their subject matter so that they can perceive the regularities and the structural properties that are so important for mathematics. Pushing forward this geometrical metaphor, a too close look at mathematical reality would miss some important aspects that can be grasped only at the appropriate distance. That this comes at the price of losing transparency of the specific object is, in our opinion, the conclusion we can draw from many of the negative results that logic has taught us in the last century.

There is an important trade‐off between the opacity of our mathematical epistemology and the expressiveness of our mathematical language. We lose local clarity to gain global understanding. That this is an obligatory passage for the increase of our understanding is an important claim that we will not try to defend here but that underlies our perspective. At this point, we can only point at where to look for a more substantial argument. For example, a simple cardinality argument shows that language can express less than what is needed for a full description of an uncountable mathematical reality, or the clear separation between truth and provability shows the limits of our axiomatic understanding of mathematics. The list could be greatly extended, but we leave this task for another occasion. Nonetheless, we can offer a moral from this reflection on the opacity of mathematical objects. The lack of transparency is not a limit of mathematical epistemology, but the proof that a little expressiveness goes a long way and that abstraction principles are fundamental aspects of our modern understanding of mathematics.

## Footnotes

1 Addressing this question in full generality, however, is beyond the scope of this paper.

2 Think about the title of Crispin Wright (1983) book *Frege*'*s Conception of Numbers as Objects*. Of course, this emphasis on objects is not the only possibility. For example, Greek mathematics was very far from being a science of objects. At that time, mathematics was centered on the notion of measure to the extent that many (but of course not all) of the philosophical concerns of that period are now quite unfamiliar to a modern eye. Historically, the view that mathematics is primarily concerned with objects is a relatively new idea.

3 The relevant notion of *sufficiency* is the one used by Linnebo in §1.5 of (Linnebo, 2018). The notion of “unproblematic” object is used here informally. The unproblematic objects are simply the elements of the base domain *D*: see below.

4
*Identity* over the base domain is the strongest equivalence relation on that domain. That does not make it the most *complex*. The unity relation in some cases is more complex.

5 Traditional Platonism, the kind of Platonism championed by Frege, is the combination of two views (see Linnebo, 2017, pp. 31–32): *object realism –* the doctrine that there are abstract mathematical objects that exist independently of our thought and language; and *truth value realism* – the view that mathematical statement have an “objective” truth value independently of our ability to establish it. It is worth noticing that one the most prominent opponents of Platonism, Hartry Field, has recently declared that the aspect of traditional Platonism that he finds more problematic is truth value realism (Field, 2016, introduction to the new edition).
